# Case report: MSI-H, EGFR mutation, and ground-glass nodules as diffuse pulmonary hematogenous metastases

**DOI:** 10.3389/fimmu.2024.1478205

**Published:** 2024-10-28

**Authors:** Liuer He, Lin Li

**Affiliations:** ^1^ Department of Oncology, Beijing Hospital, National Center of Gerontology, Institute of Geriatric Medicine, Chinese Academy of Medical Sciences, Beijing, China; ^2^ Graduate School of Peking Union Medical College, Beijing, China

**Keywords:** EGFR, MSI (microsatellite instability), GGN (ground-glass nodule), CEA, lung cancer

## Abstract

Ground-glass nodules (GGNs) are generally considered an early stage of lung cancer. The imaging characteristics and curative efficacy of multiple GGNs as metastases remain unclear. Microsatellite instability-high (MSI-H) is a biomarker for immunotherapy. The therapeutic effect and prognosis for patients with MSI-H and Epidermal Growth Factor Receptor (EGFR)-sensitive mutation stays uncertain. Here, we report a case of a lung adenocarcinoma patient presenting with ground-glass metastases, MSI-H, and EGFR-sensitive mutation and provide clinical data on the efficacy and prognosis. We describe the predictive significance of carcinoembryonic antigen (CEA) for disease progression when there is inconsistency between treatment effectiveness and CEA changes.

## Highlights

- In lung adenocarcinoma, multiple ground-glass nodules can indicate hematogenous metastases rather than synchronous multiple primary lung cancers.
- For lung adenocarcinoma patients with concurrent Microsatellite Instability-High, EGFR-sensitive mutation, and ground-glass metastatic lesions, treatment with EGFR- Tyrosine Kinase Inhibitors can achieve partial response but with a short progression-free survival.
- When a patient’s symptoms and imaging findings significantly improve after treatment, a persistently elevated carcinoembryonic antigen level suggests occult metastasis and a poor prognosis.

## Introduction

Unlike the randomly distributed solid nodules formed by hematogenous metastasis, ground-glass nodules (GGNs) are believed to be tumor cells adhering to the alveolar walls, appearing as ground-glass opacity on CT scans. Multiple GGNs in the chest are most commonly associated with synchronous multiple primary lung adenocarcinomas. MSI-H occurs in only 0.5% of lung cancers when detected by NGS (1). Research found that lung cancer patients with MSI-H gained a long-term benefit from immunotherapy (2). Here, we report a case of a lung adenocarcinoma patient presenting with multiple GGN as metastases, MSI-H, and EGFR exon 21 L858R mutation. This patient also exhibited an EGFR exon 2 A289V mutation and amplifications of other genes. EGFR A289V mutation is associated with a higher invasive phenotype and poorer prognosis (3). Previous case highlighted that a patient with stage IV lung adenocarcinoma harboring the EGFR A289V mutation achieved a sustained response of 5 months when treated first-line with Icotinib (4).

Our report aims to elucidate the imaging characteristics of GGNs as metastatic lesions of lung adenocarcinoma, the effectiveness of targeted therapy for MSI-H coexisting with EGFR-sensitive mutations, and the significance of CEA as a guide when there is a discrepancy between disease remission and CEA changes.

## Case presentation

**Figure 1:**
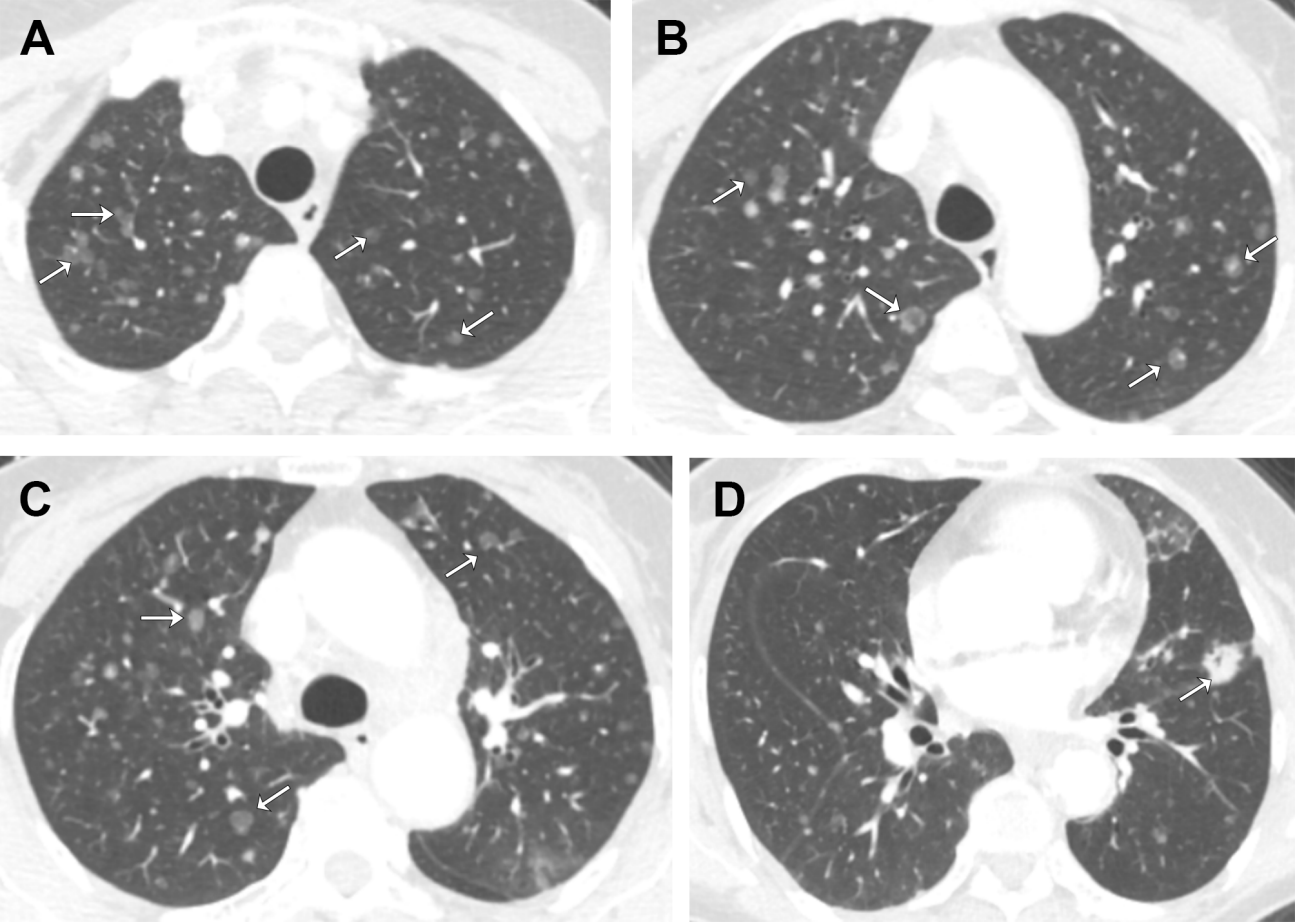
Axial contrast-enhanced chest CT images from the patient’s initial visit. Selected images from **(A)** apices, **(B)** aortic arch, **(C)** above carina, **(D)** aortic root. There are diffuse, randomly distributes ground-glass nodules in both lungs. Note that some nodules have central lucency (arrows), while others have central solid density (arrows).

A 68-year-old female patient presented with a dry cough and chest tightness, without accompanying coughing up phlegm or hemoptysis. The patient is a farmer with a medical history of hypertension and diabetes, and the patient has never smoked. The patient’s sister suffers from cervical cancer. Enhanced CT scans revealed a primary lesion in the lower lingular segment of the left upper lobe (1.5 cm x 1.4 cm, **Figure 1D**), diffuse ground-glass nodules(GGNs) as multiple pulmonary metastases in both lungs (**Figures 1A–C**), multiple enlarged lymph nodes in the mediastinum and left hilum, and left-sided pleural effusion, leading to a diagnostic thoracocentesis.

The pleural fluid pathology confirmed adenocarcinoma, originating from the lung, as confirmed by immunohistochemistry: CK7 (+++), CK20 (-), WT-1 (mesothelial+), D2-40 (mesothelial+), Desmin (mesothelial+), EMA (+++), CEA (+), TTF-1 (++), PAX8 (-), Gata3 (-), CDX2 (-), and MSH2 (+without nuclear expression loss), MSH6 (+without nuclear expression loss), MLH1 (+without nuclear expression loss), and PMS2 (+without nuclear expression loss). A 1021 panel NGS of the pleural sample showed MSI-H, EGFR EX21 p.L858R (44.4%), EGFR EX7 p.A289V (42.5%), EGFR amplification (copy number 4.4), Tumor Protein 53 (TP53) EX5 p.A159D (59.9%), along with amplifications of RAC1, GNAS, MCL1, PMS2, IKZF1, SDHA, AURKA, PDCD1LG2, CD274, SDHB, and ERCC4.

**Figure 2:**
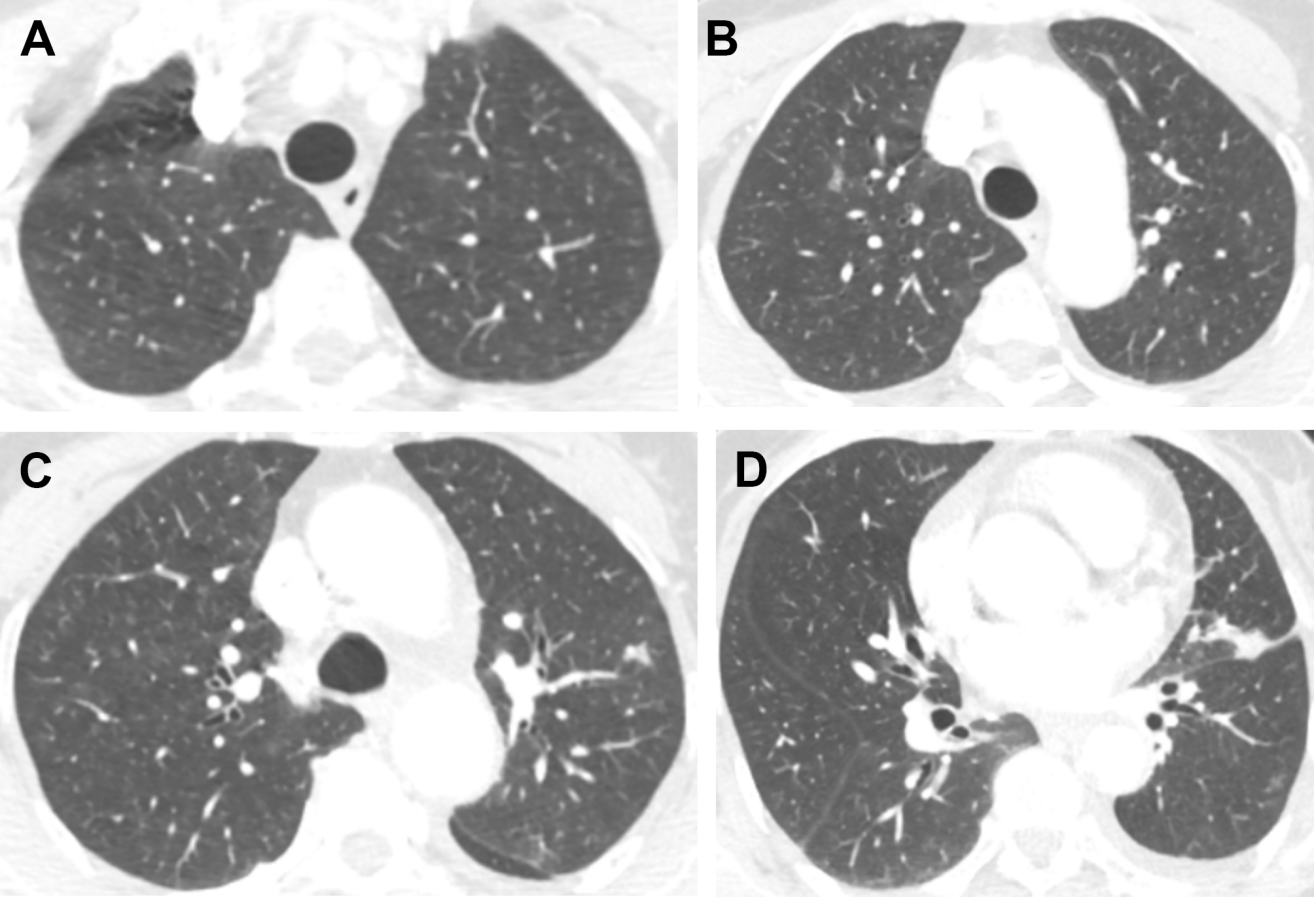
Axial unenhanced chest CT images after the treatment of EGFR TKI. Selected images from **(A)** apices, **(B)** aortic arch, **(C)** above carina, **(D)** aortic root. After targeted therapy, the diffuse ground-glass nodules in both lungs, indicative of metastasis, disappeared.

According to the CSCO guidelines, we recommended that the patient be treated with a third-generation EGFR TKI. However, due to financial constraints, the patient opted for Icotinib, a first-generation EGFR TKI, as the first line treatment (5). Then the patient was treated with Icotinib 125mg Tid. Evaluations after 2 and 4 months both showed a partial response (PR), with a significant decrease in the diffuse GGNs (**Figures 2A–C**) and a reduction in the primary lesion (**Figure 2D**) and lymph node metastatic lesions. However, despite the improvement in the patient’s symptoms, the patient’s CEA levels continued to rise, from a baseline of 9.4 ng/ml to 17.3 ng/ml, without any evidence of a secondary primary tumor on CT and symptom.

**Figure 3:**
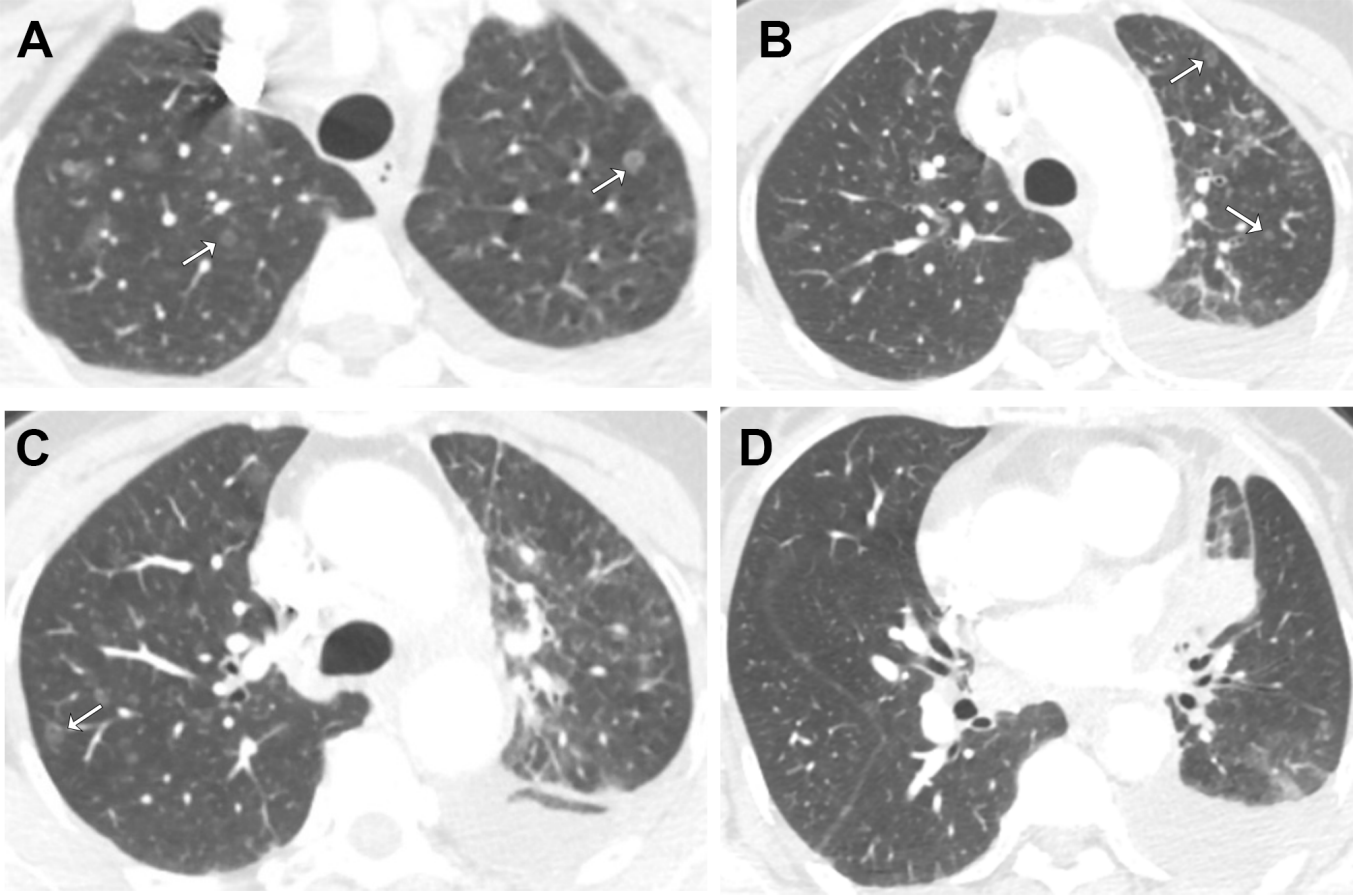
Axial contrast-enhanced chest CT images after the resistance to EGFR TKI. Selected images from **(A)** apices, **(B)** aortic arch, **(C)** above carina, **(D)** aortic root. following recurrence, multiple ground-glass nodular metastases reappeared in both lungs (arrows).

After 6 months, disease progression (PD) was noted with an increase in volume of the primary lesion, new pleural effusion, and CEA rising to 148.5 ng/ml. Then the patient received two cycles of treatment combining Icotinib 125mg Tid po with bevacizumab 400mg (7.5 mg/kg) ivgtt. However, PD was noted with recurrence of multiple GGNs in both lungs (**Figures 3A–D**), multiple new cancerous emboli, new para-aortic lymph node metastases, new metastases in bones, and CEA rising to 290.7 ng/ml. The patient developed a cough with dark red sputum and right hip pain. Another thoracocentesis was performed, and a 9-gene panel by qPCR confirmed EGFR EX21 p.L858R and EGFR EX20 p.T790M mutations. The patient was treated with osimertinib 80mg Qd. We plan to initiate immunotherapy after the patient develops resistance to osimertinib. However, the patient refused to return for follow-up examinations and discontinued osimertinib on her own after 4 months of treatment. Six months after stopping osimertinib, the patient passed away, resulting in an overall survival (OS) of 18 months.

## Discussion

Our case report firstly documents a lung adenocarcinoma patient presenting with concurrent MSI-H, EGFR-sensitive mutations, and diffuse ground-glass nodules as multiple pulmonary hematogenous metastases. There are three reasons why the pulmonary ground-glass nodules in this case are metastatic lesions from lung adenocarcinoma. First, the ground-glass nodules in this patient have well-defined borders without lobulation or spiculation, which are not consistent with the imaging features of early-stage lung adenocarcinoma. Second, the diffuse ground-glass nodules in both lungs significantly decreased or even disappeared after treatment of Icotinib. Third, after the patient developed resistance to Icotinib, diffuse ground-glass nodules in both lungs reappeared. Previous study have suggested that airway dissemination is the reason why metastatic tumors appear as ground-glass nodules on CT images (6). However, signs of aerogenous metastasis in lung cancer typically present as multiple centrilobular nodules at the ends of respiratory bronchioles, often appearing in a tree-in-bud sign. In our case, the metastatic lesions were well-defined, round nodules randomly distributed across both lungs, aligning more with the characteristics of hematogenous metastases. The patient’s CT scans revealed synchronistic solid metastatic tumors, pure GGNs and mixed GGNs, which provided radiological evidence of the tumor’s heterogeneity. Interestingly, all the nodules significantly reduced following EGFR-TKI treatment. Notably, despite the patient’s symptoms improved markedly and the treatment efficacy was evaluated as PR after treatment with Icotinib, the CEA level continuously rose without evidence of a secondary primary tumor. Typically, in tumor patients, CEA levels decrease when their symptoms significantly improve and their imaging show PR. However, in this patient, CEA levels continued to rise even after receiving Icotinib treatment, indicating the presence of occult metastases and the predictive significance of CEA for disease progression when there is inconsistency between treatment effectiveness and CEA changes (7). Levels of CYFRA 21-1 and CA125 decreased after Icotinib treatment but rose again after developing resistance, and both of them remained above the normal range throughout. NSE and proGRP followed a similar trend but stayed within the normal range the entire time. Additionally, we observed that the patient experienced hematogenous and lymph node metastases in a highly synchronous manner. Previous studies have indicated that Vascular Endothelial Growth Factor (VEGF) can simultaneously mediate hematogenous and lymphatic dissemination (8), suggesting that monitoring VEGF levels in this patient’s treatment could more sensitively reflect the disease progression.

Previous studies have reported four cases of MSI-H in a cohort of 480 lung adenocarcinoma patients (9), two of whom exhibited EGFR mutations (both Tier III mutations: EGFR p.L792F c.2374C>T and EGFR p.Q812* c.2434C>T). Both patients were male smokers with pT1N0M0-stage disease. In contrast, our case involves a female patient with no smoking history, diagnosed at stage IV (T2N2M1), carrying a Tier I mutation (EGFR p.L858R c.2573T>G). Another study reported a case of lung cancer with concurrent MSI-H and EGFR Exon19del (10), though the patient’s pathological subtype was pulmonary enteric adenocarcinoma (PEAC). Given that the incidence of MSI-H in colorectal cancer is about 15% and that over 50% of the tumor in this PEAC patient showed intestinal differentiation, the occurrence of MSI-H in this case may be related to histological and molecular characteristics similar to those of intestinal adenocarcinoma. In both studies, chest imaging did not reveal atypical lung cancer signs. Ground-glass nodules (GGNs) are generally considered an early manifestation of lung adenocarcinoma, but in our case, the patient presented with diffuse GGNs in both lungs at the time of initial diagnosis. Previous hypotheses have suggested that airway dissemination could explain the GGN appearance in metastatic lung cancer. However, the patient’s chest CT, which showed randomly distributed GGNs across both lungs rather than concentrated at the terminal bronchioles, is more consistent with hematogenous metastasis. In this case, The presence of both MSI-H and EGFR exon 21 L858R mutations in this patient provides pathological evidence of tumor heterogeneity. Exon 2 is located outside the tyrosine kinase domain (exons 18, 19, 20, and 21) that EGFR TKIs primarily target. Therefore, the EGFR EX7 p.A289V mutation may be the reason for the patient’s relatively short PFS and primary resistance to EGFR TKI treatment. Moreover, the amplification of TP53 and EGFR can promote primary resistance to targeted therapy, contributing to the patient’s relatively short PFS in EGFR TKI treatment (11, 12). In approximately 50-60% of cases of acquired resistance, the resistance to EGFR TKI is due to the development of the EGFR T790M mutation. This mutation occurs in exon 20 of the EGFR gene and alters the structure of the tyrosine kinase domain, reducing the inhibitory effect of the TKIs on the EGFR receptor. Therefore, we believe that the EGFR EX20 p.T790M mutation is the cause of the patient’s lung cancer recurrence and acquired resistance. Above reasons partially explains the shorter progression-free survival (Progression-Free Survival=6 months) observed in this patient. Furthermore, previous studies have found that lung cancer patients with MSI-H can benefit from immunotherapy, and immune checkpoint inhibitors should be considered in the treatment of this patient (2).

## Data Availability

The raw data supporting the conclusions of this article will be made available by the authors, without undue reservation.
